# Prokineticin 2 and Cytokine Content in the Synovial Fluid of Knee Osteoarthritis and Traumatic Meniscal Tear Patients: Preliminary Results

**DOI:** 10.3390/jcm12134330

**Published:** 2023-06-28

**Authors:** Marco Turati, Silvia Franchi, Marco Crippa, Laura Rizzi, Luca Rigamonti, Paola Sacerdote, Simone Daniel Gatti, Massimiliano Piatti, Giulia Galimberti, Daniele Munegato, Giada Amodeo, Robert J. Omeljaniuk, Giovanni Zatti, Antonio Torsello, Marco Bigoni

**Affiliations:** 1 Orthopedic Department, Fondazione IRCCS San Gerardo dei Tintori, 20900 Monza, Italy; marco.turati@unimib.it (M.T.);; 2 School of Medicine and Surgery, University of Milano-Bicocca, 20900 Monza, Italy; 3 Transalpine Center of Pediatric Sports Medicine and Surgery, University of Milano-Bicocca, 20900 Monza, Italy; 4 Department of Paediatric Orthopedic Surgery, Hospital Couple Enfants, Grenoble Alpes University, 38400 Grenoble, France; 5 Department of Pharmacological and Biomolecular Sciences, University of Milan, 20129 Milan, Italy; silvia.franchi@unimi.it (S.F.);; 6 Department of Orthopedic Surgery, Policlinico San Pietro, 24036 Ponte San Pietro, Italy; 7 Department of Biology, Lakehead University, Thunder Bay, ON P7B 5E1, Canada

**Keywords:** meniscus, osteoarthritis, cytokines, knee, prokineticins, synovial

## Abstract

Knee osteoarthritis (OA) is a chronic degenerative inflammatory-based condition caused by a cascade of different intra-articular molecules including several cytokines. Among the cytokines, prokineticins (PKs) have recently been identified as important mediators of inflammation and pain. This observational study examined the potential involvement of PK2 in degenerative or traumatic knee disease. Fifteen patients presenting knee osteoarthritis (OA group) and 15 patients presenting a traumatic meniscal tear (TM group) were studied. Synovial fluid samples from affected knees were assessed for PK2, IL-10, and TNF-α using the ELISA method. At a long-term follow-up (minimum 5 years, mean = 6.1 years), patients in the TM group underwent clinical re-evaluation with PROMs (Tegner Activity Scale, IKDC, Lysholm, SKV); in addition, X-ray visualization was used to assess the presence of secondary OA. PK2 was detected in synovial fluids of both TM and OA patients and the levels were comparable between the two groups, while IL-10 levels were significantly greater in the OA group than those in TM patients. PK2 levels correlated with those of IL-10. PK2 levels were greater in blood effusions compared to clear samples, did not differ significantly between sexes, nor were they related to differences in weight, height, or injury (meniscal laterality, time since dosing). No correlation was found between PROMs and radiological classifications in patients in the TM group at final follow-up. These data are the first observations of PK2 in synovial fluid following traumatic meniscus injury. These findings suggest possible further prognostic indices and therapeutic targets to limit the development of secondary OA.

## 1. Introduction

Knee osteoarthritis (OA), the most common form of arthritis [1,2], is a debilitating chronic degenerative disease affecting articular cartilage that progressively erodes and causes pain, difficulty in movement, and, in more severe cases, joint deformation. It presents in patients of advanced age [1], but early-onset OA may develop as a consequence of knee trauma or surgery. The incidence of OA will likely increase in the future in particular due to increases in the average age as well as prevalence of obesity in the first world [3]. It is recognized that the inflammatory environment within the joint consequent to traumatic lesions, such as meniscal tear or anterior cruciate ligament (ACL) rupture, strongly influences the healing process and shapes long-term articular damage [4,5,6]. Elevated levels of inflammatory cytokines in synovial fluid are associated with many joint diseases, including OA [7,8] and traumatic lesions of the knee [8]; however, the pathophysiological role of these mediators remains undefined.

The identification and characterization of biological markers in synovial fluid, such as cytokines, is becoming an important strategy for patient profiling and as a prognostic marker for disease progression [9].

Among cytokines, prokineticins (PKs) were recently identified as important mediators of inflammation and pain [10]. This chemokine family includes two ligands, PK1 and PK2, and two g-protein coupled receptors, PKR1 and PKR2. PK2 is usually upregulated in inflamed tissue, thereby promoting inflammation and nociceptive sensitization [11,12]. Moreover, elevated PK2 levels were recently found in the peripheral blood of patients with inflammatory diseases, such as multiple sclerosis [13] and psoriasis [14]. Both PKs and PKRs are widely present in different human tissues, such as immune and endothelial cells; moreover, their presence has also been described in regions of the nervous system associated with pain, such as brain areas and spinal cord and peripheral nerves, where both neurons and glial cells can produce or express them.

In joints, different cells may express PKRs and produce PK2 [15,16]. Infiltrating granulocytes, synovial cells including synovial fibroblasts and macrophages, and inflammatory cells are certainly an important source of PK2 release in synovial fluid [15]. The number of these cells and their activation state varies according to the phase of disease development. For example, infiltrating granulocytes are reduced in the synovial tissue of chronic arthritis models compared to acute arthritis, suggesting that the source of PK2 could also change during the progression of pathology. In addition, it was recently suggested that PK2 could have dualistic effects in arthritis; specifically, pro- and anti-inflammatory influences appear to depend on the effector cell type, phase of disease, and the presence or absence of additional cytokines [15].

In particular, the role of prokineticin 2 (PK2) was studied in the pathogenesis of different forms of arthritis in both preclinical models and in humans. In a murine model of collagen-induced arthritis (CIA) [16], PK2 and PKR2 gene expression levels were significantly elevated in the joints of CIA mice; this over-expression correlated with arthritis severity. These data were confirmed in a recent paper [17] that demonstrated that not only PK2 and PKR2 but also PKR1 were overexpressed in inflamed joints where they contributed to the progression of chronic arthritic inflammation by increasing the expression of proinflammatory cytokines such as IL-1β and TNF-α.

In addition, both PK2 and PKRs were expressed in the synovial tissue of rheumatoid arthritis (RA) and osteoarthritis (OA) patients [15]. RA patients showed higher levels of PK2 in synovial fluid in comparison to that of OA patients, but the real role of PK2 has not yet been defined.

Given the current understanding of the role of PK2 in inflammation and pain, we hypothesize that PK2 may also be detected in traumatic knee injuries such as meniscal tears; there are currently no published data on PK levels in the synovial fluid of individuals with traumatic knee injuries.

Consequently, we evaluated the levels of synovial fluid PK2 in two different cohorts of patients including a group with acute meniscal tears and a group with chronic knee osteoarthritis. In addition, we also assessed synovial fluid levels of TNF-α, a pro-inflammatory molecule involved in cartilage degradation in OA, and of those of IL-10, an anti-inflammatory cytokine which typically increases after trauma and which is antagonistic to TNF-α [18].

Lastly, in the traumatic group, we evaluated a potential association between cytokine levels, PROMS (*Patient-Reported Outcome Measurements*), and X-ray visualization of the joint at least 5 years after trauma. To date, there are no previous studies which have undertaken a retrospective analysis between type and levels of cytokines in synovial fluid following meniscal injury, and the presence at a distance of at least 5 years of OA. Overall, we assessed the possibility of a prognostic role and therapeutic target of cytokines.

## 2. Materials and Methods

### 2.1. Subjects

Thirty patients were enrolled between 2017 and 2018 and resolved into one group with knee OA as defined by the American College of Rheumatology’s criteria and with at least grade 2 on Kellgren–Lawrence radiological classification [19] (OA group, *n* = 15) and a second group presenting isolated traumatic meniscal tears without OA (Traumatic Meniscal, TM group, *n* = 15).

The OA patients had no history of knee infectious or inflammatory disease, previous surgery, ligamentous/meniscal tears, or intra-articular steroid or hyaluronic acid injections in the previous 12 months. TM patients presented isolated meniscal tears, confirmed by a senior orthopedic surgeon, in consideration of patient history, objective examination, MRI imaging, and arthroscopic confirmation of the lesion. Exclusion criteria included (i) previous lesions or intervention to the investigated or contralateral knee, (ii) presence of bony or ligamentous lesions, (iii) grade > 1 of chondropathy according to the Outerbridge classification [20], (iv) arthritis or chronic inflammatory diseases, (v) polypharmacotherapy (>3 drugs), (vi) osteochondral autograft transfer, (vii) absence of joint effusion, and (viii) intra-articular injection of drugs.

OA patients’ synovial fluids were collected during clinical evaluations in the specialist outpatient setting of our Orthopedic Department, whereas TM patient samples were taken either in the emergency department or during arthroscopic surgery.

In order to evaluate the effect of time between traumatic injury and synovial fluid sampling, TM patients were resolved into two groups according to the time elapsed between the injury and fluid collection, including a Sub-acute group (SA, 3 to 90 days after injury) as well as a Chronic group (C, greater than 90 days after injury).

### 2.2. Clinical and Radiological Follow-Up after 5 Years for Meniscal Tears

TM patients were clinically and radiographically evaluated no less than five years post-arthroscopic surgery.

In order to assess functionality of the investigated knee, Patient-Reported Outcome Measurements (PROMs) were performed (*n* = 8), which included (i) Lysholm knee questionnaire (0–100, score based on 8 different symptoms to provide knee function) [21], (ii) Tegner Activity (0–10, regarding the patient’s activity level, before and after the injury) [22], (iii) IKDC 2000 Subjective Knee Evaluation Form (0–100, global score about clinical, activity, and function) [23], as well as a (iv) Simple Knee Value [24] (0–100, subjective patient-given rating of knee function).

Weight-bearing X-rays were performed in antero-posterior (AP), latero-lateral (LL), and Rosenberg projections [25] (i.e., a postero-anterior projection with 45° flexed knee and rays on the lower pole of the patella and directed 10° caudally) according to a standardized protocol [26].

The presence and degree of OA were assessed with the Kellgren–Lawrence radiographic classification (grades 0 to 4, where 0 = healthy knee) [19].

All patients were informed about the study protocol and signed written informed consent for the use of the samples taken. The study protocol was previously approved by the Ethics Committee and was conducted in accordance with the guidelines in the WMA Declaration of Helsinki.

### 2.3. Synovial Fluid Collection and Storage

Synovial fluid sampling was performed after sterile preparation with chlorhexidine using the medial or superolateral access with a 20-gauge needle without any lavage [27]. Synovial fluids were transferred into 15 mL sterile tubes, stored in ice, and transferred to the laboratory where they were centrifuged at 3000× *g* to eliminate cells and other debris. Supernatants were frozen and stored at −80 °C until assayed [28,29,30].

### 2.4. PK2 and Cytokine Analysis

The levels of PK2 in synovial fluid were quantified using human PK2/PROK2 enzyme-linked immunosorbent assay (ELISA) kit (cat. n. LS-F5959; LifeSpan Biosciences, Inc., Seattle, WA, USA) according to manufacturer instructions. TNF-α as well as IL-10 were also measured in synovial fluid using a specific sandwich ELISA according to the manufacturer’s instructions (R&D Systems, Minneapolis, MN, USA). For all patients, the analysis was performed at the end of the enrollment period.

All samples were run in duplicate. Levels are expressed as pg/mL.

### 2.5. Statistics

The Shapiro–Wilk test, initially used to evaluate data distribution, revealed that the data were not normally distributed. Consequent statistical analyses involved non-parametric tests. Two types of analyses were conducted including (i) a comparison between two (or more) groups, and (ii) correlation analysis between two variables.

In analyzing the two groups, the Mann–Whitney test was applied, whereas the nonparametric ANOVA test, the Kruskal–Wallis test, was used to analyze comparisons among multiple groups. Correlation analysis between two variables was assessed using the Spearman nonparametric test. For each test, the significance level was set at *p* < 0.05. Both descriptive analyses and statistical analyses were conducted using the computer program GraphPad Prism 9.

All the results are expressed as Mean ± SD (Standard Deviation).

## 3. Results

OA patients, including 7 males and 8 females, ranged in age from 52 to 90 years (mean = 72.9 ± 9.5 years) and were considered Chronic (C). Regarding the macroscopic characteristics of the synovial fluid (clear or hematologic), synovial fluid samples of 11 patients were found to have hydrarthrosis, whereas four patient samples presented hemarthrosis (Table 1).

TM patients ranged in age from 24 to 57 years (mean = 43.7 ± 9.6 years), were males, and played sports at various levels of training and competition; seven were considered sub-acute (SA), whereas eight were considered Chronic (C). Twelve patients presented medial meniscus tears, while three patients presented lateral meniscus tears. All TM patients underwent the same standardized arthroscopic procedure of partial selective meniscectomy.

At the five-year follow-up, the eight Chronic (C) TM patients underwent re-evaluation via PROMs and showed great variability among the results; to illustrate, IKDC and SKV were similar (69.55 ± 16.42 and 71.25 ± 13.30, respectively), whereas the Lysholm scores were 80.63 ± 10.35. Previously injured patients had 5.25 ± 2.71 Tegner scores, while after trauma and meniscectomy, they worsened, with Tegner scores of 4.50 ± 2.00 (Table 2).

X-ray evaluation was performed on six patients at a 5-year follow up; the Kellgren–Lawrence grade is reported in Table 2.

The levels of PK2, TNF-α, and IL-10 measured in synovial fluid from all groups are reported in Table 3 (OA patients) and Table 4 (TM patients).

### 3.1. Effect of the Type of Injury on PK2 and Cytokine Levels

**Figure 1:**
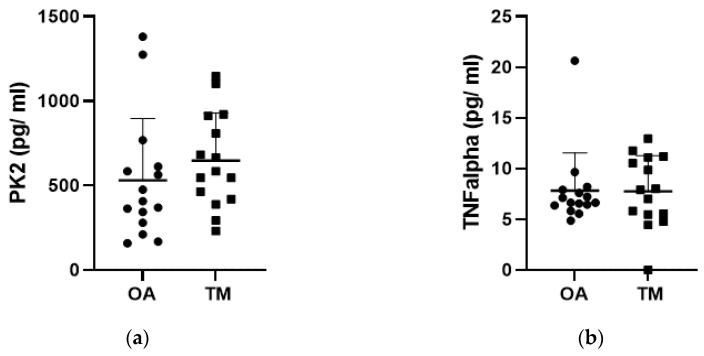

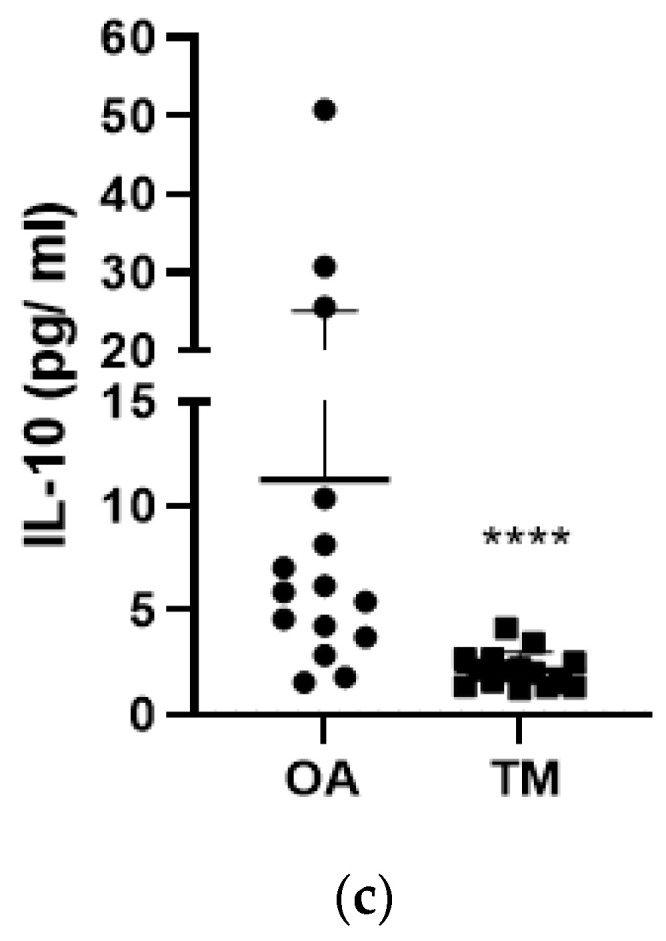
PK2 and cytokine content in synovial fluid. PK2 (**a**), TNF alpha (**b**), and IL-10 (**c**) in the synovial fluid of knee osteoarthritis (OA) and Traumatic Meniscal (TM) patients. Solid circles and solid squares represent PK2 and cytokines synovial fluid content of each single patient in OA (circle) and TM (square) group. The levels of biomolecules are expressed as pg/mL. **** *p* < 0.0001 Mann–Whitney test.

Synovial fluid samples from all patients contained measurable concentrations of PK2 (Figure 1a). However, no statistically significant differences were discerned in PK2 levels in the OA group compared with the TM group. Similarly, TNF-α levels were not significantly different between the two groups (Figure 1b). Unlike PK2 and TNF-α, IL-10 concentrations were significantly greater in the OA group compared with those in the TM group (*p* < 0.0001, Figure 1c).

### 3.2. Effect of Time between Injury and Synovial Fluid Sampling on PK2 and Cytokine Levels

**Figure 2:**
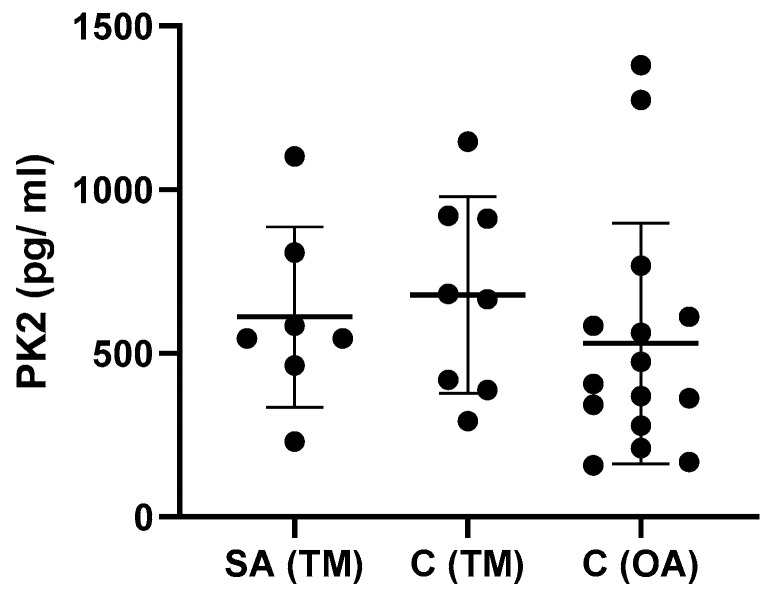
Effect of elapsed time between traumatic injury and synovial fluid sampling on PK2 levels. PK2 levels (pg/mL) were reported in Sub-acute group (SA, synovial fluid sample collected from 3 to 90 days after injury) and in Chronic group (C, synovial fluid sample collected greater than 90 days after injury) belonging to both TM and OA groups. Solid circles represent PK2 synovial fluid content of each single patient.

PK2 levels were plotted as a function of the time elapsed between traumatic injury and synovial fluid sampling (Figure 2). The data indicate no significant difference in PK2 levels between the subacute (SA) and chronic (C) traumatic knee injury groups, nor was there a significant difference in PK2 levels between the OA group and TM group at any time. Time kinetics were not shown for IL-10 or TNF-α (Table 3 and Table 4).

In addition, meniscal laterality does not appear to influence the levels of PK2 and cytokines in TM patients (Table 4).

### 3.3. Effect of Type of Samples on PK2 TNF-α and IL-10 Levels

**Figure 3:**
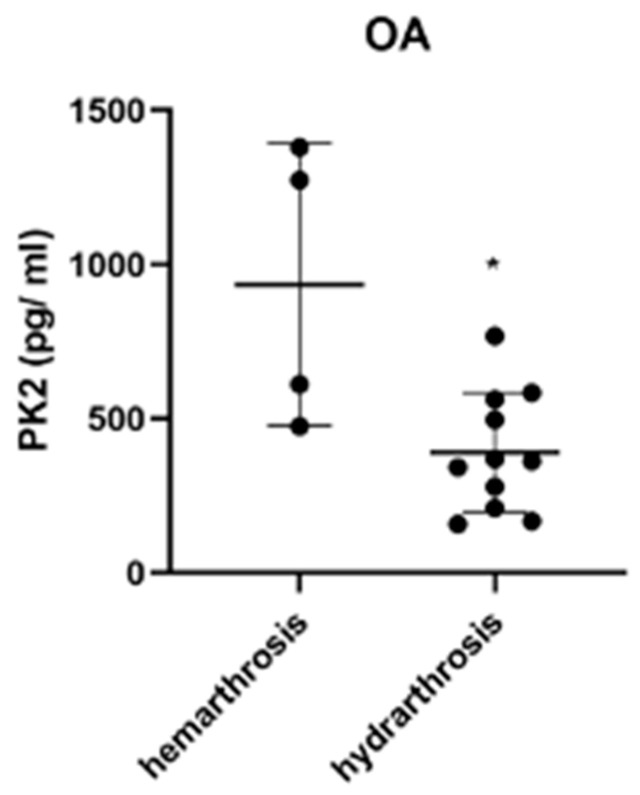
Effect of synovial fluid quality on PK2 levels. PK2 levels were evaluated (pg/mL) in hematologic (hemarthrosis) and clear (hydrarthosis) synovial fluid samples of Knee Osteoarthritis patients (OA group). * *p* < 0.05 Mann–Whitney test. Solid circles represent PK2 synovial fluid content of each single patient.

We also assessed the effects of sample quality on PK2 levels. In the case of OA patients, PK2 levels were significantly (*p* < 0.05) greater in hematologic (hemarthrosis) samples compared with clear (hydrarthosis) samples (Figure 3). By comparison, there was no significant influence of sample quality on the levels of the two cytokines (Table 3 and Table 4).

### 3.4. Effect of Gender and Age on PK2 Levels

**Figure 4:**
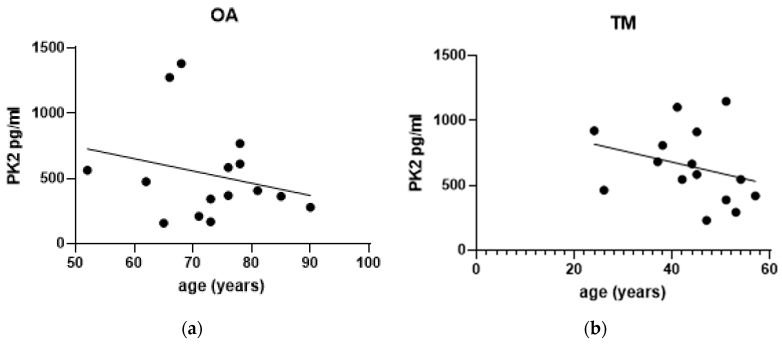

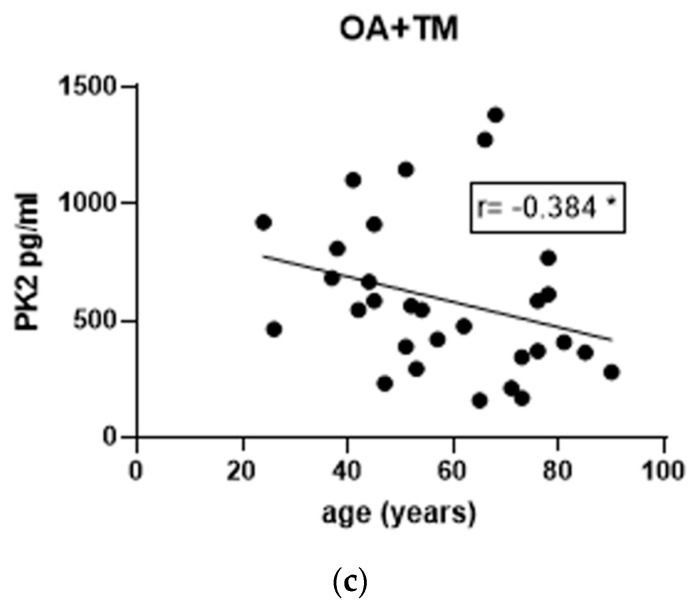
Correlation between age of patients and PK2 levels. The effect of age on PK2 levels in the synovial fluid was evaluated in Knee Osteoarthritis (OA group; (**a**)) and in Traumatic Meniscal (TM group; (**b**) patients and in the pooled OA+TM population (**c**). Correlations were investigated using the non-parametric Spearman rank correlation coefficient test. R and significant p-value are reported in figure; * *p* < 0.05. Solid circles represent values of each single patient.

Although the TM group represented only one gender, both genders were represented in the OA group; nonetheless, there was no significant difference in PK2 levels between genders (Table 3 and Table 4). With respect to the influence of age, data pooled together from the OA and TM groups revealed a significant inverse correlation between age and PK2 levels (r = −0.384, *p* = 0.0361) (Figure 4c). Within each of the two groups, there was no significant correlation between age and PK2 levels; nonetheless, there was an inverse (non-significant) trend of age in the TM group (*p* = 0.0796). We did not detect any gender (Table 3) or age influence on the levels of the two cytokines.

### 3.5. Correlations of IL-10 and TNF-α Levels with PK2

**Figure 5:**
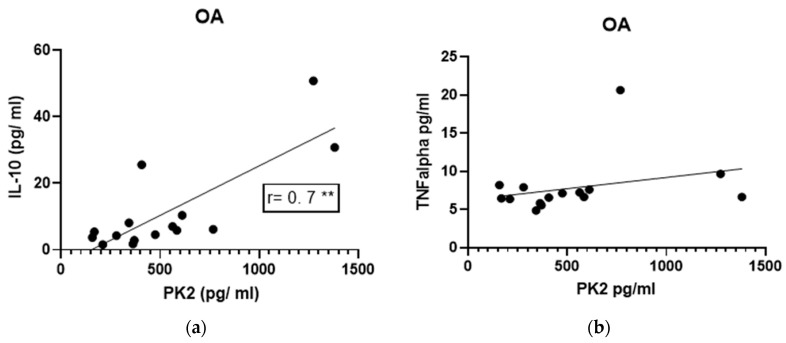

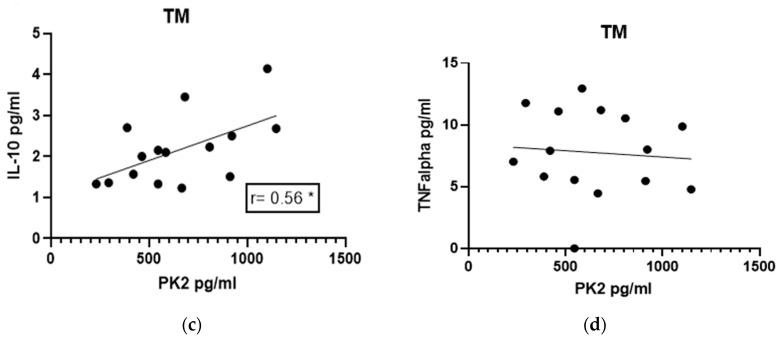
Correlation between cytokines and PK2 measured in the synovial fluid. PK2 levels in the synovial fluid were correlated to that of IL-10 and TNFalpha measured in Knee Osteoarthritis (OA group; (**a**,**b**)) and in Traumatic Meniscal (TM group; (**c**,**d**)) groups. Correlations among molecules were investigated using the non-parametric Spearman rank correlation coefficient test. r and significant *p*-value are reported in figure; * *p* < 0.05; ** *p* < 0.01. Solid circles represent values of each single patient.

We considered potential coordinate relationships in levels among these molecules in the two patient groups. There was a significant positive correlation between PK2 and IL-10 levels in the OA group (*p* = 0.0048, r = 0.70, Figure 5a), as well as in the TM group (*p* = 0.0294, r = 0.56, Figure 5c). There were no correlations between TNF-α and PK2 levels in either group (Figure 5b,d).

### 3.6. Correlations between Cytokine Levels and PROMS and X-ray

Eight TM patients received a follow-up examination at least five years after their initial intake and synovial fluid sampling and analysis, and results of the follow-up examinations were compared to the initial levels of PK2 or cytokines. No correlations were found between initial PK2 levels or cytokines and either (i) Tegner Activity Scale pre-injury, (ii) Tegner Activity Scale post-injury, (iii) Lysholm, (iv) IKDC and SKV, or (v) the radiological classification for OA (Kellgren–Lawrence). All patients developed OA prior to the follow-up examination; 50% of patients had initial OA (KL = 1), while the other 50% had clear OA (KL ≥ 2).

## 4. Discussion

Prokineticins (PK2) were detected in the synovial fluid of patients with knee osteoarthritis as well as in patients with traumatic meniscal tears. Although the presence of PK2 was recently reported by Noda et al. [15] in OA, no other author to date has described its presence in acute knee injury; in this manuscript, for the first time, PK2 levels were detected in the synovial fluid of traumatic meniscal tears. PK2 is usually over-expressed in damaged tissue where it promotes inflammation. In the joint, both immune system cells and synoviocytes produce PK2, which may contribute to the recruitment of other infiltrating cells that express PK2 receptors at the site of injury, thereby sustaining a pro-inflammatory loop [11,12]. Moreover, PK2 is involved in nociceptive sensitization, a key element of both studied knee pathologies. It is known that PK2 can directly stimulate nociceptors, interacting with PKRs, or induce the release of pro-algogen mediators like calcitonin gene-related peptide-CGRP, substance P-SP, or cytokines which can, in turn, sensitize nociceptors [11,12]. Therefore, these two knee disorders, although one is chronic-degenerative and the other is acute-traumatic, seem to share this inflammatory mediator pattern. However, despite these considerations, the specific role(s) of PK2 and how it fits within the pathophysiology of the disease remains unclear.

Furthermore, we evaluated a possible difference in PK2 levels between the two groups, degenerative and traumatic. Although we initially assumed that PK2 levels in the TM group would be greater because of the acute nature of this condition, the data do not support this assumption, as PK2 levels did not differ between the OA and TM groups.

Nonetheless, there is a significant difference between the two groups in IL-10 content; specifically, IL-10 concentrations were greater in the OA group than in the TM group. IL-10 is an anti-inflammatory molecule, antagonistic to pro-inflammatory influences, and consequently is chondroprotective. Increased levels of IL-10 indicate a pronounced anti-inflammatory component in degenerative injuries; by contrast, lower concentrations of IL-10 are reflective of the pro-inflammatory environment following traumatic injuries, which might contribute to post-traumatic OA onset. Support for this hypothesis was recently reported by Barker et al., wherein low serum IL-10 concentrations were found in patients with post-traumatic OA and in patients with severe OA (grade 4 according to Kellgren–Lawrence classification) [31].

The correlation between PK2 and IL-10 is interesting in that it suggests possible roles for PK2. While the anti-inflammatory role of IL-10 is already well-described [32,33], the role of PK2 might be both pro- and anti-inflammatory [15]. A direct link between these two molecules supports the idea that they might act coordinately; to illustrate, an increase in PK2 activity, which might promote inflammation, may be attenuated by an increase in IL-10 levels. However, concentrations of PK2 are not correlated with TNF-α. TNF-α is one of the main pro-inflammatory molecules which activate catabolic pathways and metalloproteinases that increase cartilage damage [34].

In our sample, no differences emerged between Acute and Chronic patients, with PK2 values remaining constant over time in the TM group; this finding suggests a sustained and prolonged PK2 synthesis and release into the synovial fluid. In addition, no alterations were observed in cytokine content in the same samples. These findings differ from the literature, wherein an increasing pattern of TNF-α in patients with traumatic knee injuries has been well described [35], suggesting that it not only contributes to the initial injury but also promotes the subsequent development of PTOA due to the imbalance between pro-inflammatory and anti-inflammatory molecules. Elevated concentrations of IL-10 have also been found in acute knee traumatic pathological conditions; by contrast, lower levels of IL-10 were found in the synovial fluid of traumatic patients in chronic conditions [32,36]. Those finding suggest that this cytokine is activated in the initial hours following injury in an effort to promote repair immediately and counteract pro-inflammatory molecules, thereby antagonizing the effect of metalloproteinases and promoting collagen type II deposition [37]. The higher IL-10 levels that we have found in chronic OA patients could be due to the nature of the pathology itself, that is, a progressive degenerative condition affecting patients for a very long period of time, not comparable to the chronic condition of traumatic patients like TM and ACL patients (starting from 3 months after trauma). It could be hypothesized that over time, different macrophage subtypes (M1 and M2 macrophage) might be responsible for IL-10 production; in detail, M1 might be activated in the first phase of damage and then M2 differentiated macrophages might sustain IL-10 production in a continuous ant-inflammatory effort to counteract degeneration [38,39].

Our data also show that PK2 levels are greater in hematic (hemarthrosis) rather than in serous (hydrarthrosis) synovial effusion. By comparison, high levels of PK2 have recently been found in the peripheral blood of patients with inflammatory diseases [13,14]. An increase in blood supply leads to higher levels of infiltrating granulocytes and inflammatory cells, which produce PK2 [15,16]. Blood effusion can lead to an increased risk of OA, due to catabolic molecules, and is well-documented in the case of hemophiliac patients with frequent blood effusions [40].

Comparison of PK2 levels with age in all subjects suggests that higher production of the molecule is present in younger patients with knee joint problems, while over the years this aspect is decreased. Again, this is unique within the literature, because for the first time it was possible to observe possible different PK2 kinetics in relation to age. Unlike what was seen for PK2, IL-10 and TNF-α did not show a correlation between cytokine levels and age of patients, which is consistent with previous reports [33].

Furthermore, although being female is an additional risk factor for knee OA, this does not seem to be related to intra-articular cytokine levels, as there was no apparent difference between males and females.

This study is the first attempt to consider a possible association between cytokine levels in meniscal tears and clinical–functional outcomes after a long-term follow-up of five years; however, we found no correlation between cytokine levels and clinical outcomes. However, it must be remarked that these correlations were performed only in a small cohort of our patients, and it would be possible that with higher numbers, different results may be observed. Radiological assessment at five years of follow-up of the TM group showed no correlation between Kellgren–Lawrence classification and the examined biomarkers. Signs of knee osteoarthritis were present in all patients: early-OA in 50% of patients (Kellgren–Lawrence classification score equal to 1), but advanced OA in the remaining 50% (Kellgren–Lawrence classification score greater than or equal to 2) [41]. In the latter case, clear osteophytes, both at the margins and especially intercondylar, and a reduction in the inter-articular space were clearly visible. We note that our results are consistent and congruent with the literature. In a study by Jomha et al. [42], a prevalence of secondary OA of 72% was found at a follow-up of 7 years, while in another study by Seon et al. [43], this value was 43% at a follow-up of 11.2 years.

There are limitations in this study, including the small sample size of 30 patients, with the individual disease groups of only 15 patients each. Moreover, the TM group was not sufficiently heterogeneous; that is, sex (all patients were male), age (all patients were young), and type of effusion (all samples were hydrarthrosis). In addition, as commonly reported in this type of study, there is considerable variation in cytokine levels.

In conclusion, in spite of several limitations, the results of our study are interesting and suggest clinical implications but require further investigation. Certainly, more patient recruitment, extended over time, is necessary, as is a more rigorous characterization of patient subjects.

## 5. Conclusions

In conclusion, our study emphasizes that the environment within the injured knee joint is characterized by an inflammatory pattern sustained by different cooperating molecules, now including prokineticins (PK2), detected both during traumatic and chronic-degenerative pathologies.

In recent years, attention regarding the knee joint microenvironment following traumatic pathology has grown exponentially; our study is useful in that it suggests prognostic and population stratification perspectives and implies potential therapeutic targets.

Further studies are needed to test the hypotheses proposed, given all the limitations highlighted above. It will be useful to evaluate the role of PK2s in other traumatic knee pathologies, such as anterior cruciate ligament (ACL) rupture either associated or not with meniscal injury. Finally, it will be interesting to assess potential correlation of PK2 with other molecules, such as IL-6, IL-8, IL-1beta, Metalloproteases, or Resolvins, in order to understand the different pathways that link all these molecules in this complex microenvironment.

## Figures and Tables

**Table 1:** Patients’ characteristics. Continuous variables are reported as mean ± Standard Deviation (SD), while for categorical variables, both absolute and percentage values of the population are reported.

	OA Group	TM Group	Total
**Gender**			
Male	7 (46.67%)	15 (100%)	22 (73.33%)
Female	8 (53.33%)	0 (0%)	8 (26.67%)
**Age**			
Mean Age (Years)	72.9 ± 9.5	43.7 ± 9.6	58.3 ± 17.59
**Timing**			
Subacute (SA)	/	7 (46.67%)	7 (23.33%)
Chronic (C)	15 (100%)	8 (53.33%)	23 (76.67%)
**Synovial Fluid**			
Hydrarthrosis	11 (73.33%)	15 (100%)	26 (86.67%)
Hemarthrosis	4 (26.67%)	0 (0%)	4 (13.33%)
**Laterality**			
Medial Meniscus	/	12 (80%)	12 (80%)
Lateral Meniscus	/	3 (20%)	3 (20%)

**Table 2:** PROMs and X-ray scores. PROMs are reported as mean ± Standard Deviation (SD), whereas X-ray values are reported as percentage of patients for each level of Kellgren–Lawrence classification.

	Score
**PROMs**	
Tegner—pre-injury	5.25 ± 2.71
Tegner—after injury	4.50 ± 2.00
Lysholm	80.63 ± 10.35
IKDC	69.55 ± 16.42
SKV	71.25 ± 13.30
**RX**	
Kellgren–Lawrence	0 = 0%
	1 = 50%
	2 = 50%
	3 = 0%
	4 = 0%

**Table 3:** Knee Osteoarthritis patient group (OA group): means (pg/mL) and Standard Deviations (SDs) for the 3 different molecules dosed are shown.

	PK2	IL-10	TNF-α
**Gonarthrosis**	530.3 ± 367.1	11.26 ± 13.81	7.813 ± 3.733
**Gender**			
Male	588.6 ± 377.8	11.85 ± 11.65	6.689 ± 0.576
Female	479.3 ± 375.2	10.74 ± 16.26	8.798 ± 5.022
**Synovial Fluid**			
Hemarthrosis	935.6 ± 458.0	24.12 ± 21.02	7.755 ± 1.329
Hydrarthrosis	391.1 ± 193.2	6.584 ± 6.643	7.835 ± 4.357

**Table 4:** Meniscal tear group (TM group): means (pg/mL) and Standard Deviations (SDs) for the 3 different molecules dosed are shown.

	PK2	IL-10	TNF-α
**Meniscal tear**	647.2 ± 281.0	2.15 ± 0.843	8.314 ± 2.885
**Timing**			
SA	611.4 ± 275.6	2.182 ± 0.94	9.502 ± 2.733
C	678.6 ± 300.7	2.12 ± 0.81	7.423 ± 2.828
**Laterality**			
Medial Meniscus	626.4 ± 296.3	2.13 ± 0.94	7.888 ± 3.057
Lateral Meniscus	730.8 ± 238.4	2.24 ± 0.25	9.873 ± 1.646

## Data Availability

The data presented in this study are available on request from the corresponding author. The data are not publicly available due to privacy restrictions.
